# The Guaymas Basin Subseafloor Sedimentary Archaeome Reflects Complex Environmental Histories

**DOI:** 10.1016/j.isci.2020.101459

**Published:** 2020-08-15

**Authors:** Gustavo A. Ramírez, Luke J. McKay, Matthew W. Fields, Andrew Buckley, Carlos Mortera, Christian Hensen, Ana Christina Ravelo, Andreas P. Teske

**Affiliations:** 1 Department of Marine Sciences, University of North Carolina at Chapel Hill, Chapel Hill, NC, USA; 2 College of Veterinary Medicine, Western University of Health Sciences, Pomona, CA, USA; 3 Department of Land Resources and Environmental Sciences, Montana State University, Bozeman, MT, USA; 4 Center for Biofilm Engineering, Montana State University, Bozeman, MT, USA; 5 Department of Microbiology and Immunology, Montana State University, Bozeman, MT, USA; 6 Instituto de Geofisica, Universidad Nacional Autónoma de México, Coyoacán, México; 7 GEOMAR Helmholtz Centre for Ocean Research, Kiel, Germany; 8 Ocean Sciences Department, University of California at Santa Cruz, Santa Cruz, CA, USA

**Keywords:** Earth Sciences, Biogeochemistry, Microbiology

## Abstract

We explore archaeal distributions in sedimentary subseafloor habitats of Guaymas Basin and the adjacent Sonora Margin, located in the Gulf of California, México. Sampling locations include (1) control sediments without hydrothermal or seep influence, (2) Sonora Margin sediments underlying oxygen minimum zone water, (3) compacted, highly reduced sediments from a pressure ridge with numerous seeps at the base of the Sonora Margin, and (4) sediments impacted by hydrothermal circulation at the off-axis Ringvent site. Generally, archaeal communities largely comprise Bathyarchaeal lineages, members of the Hadesarchaea, MBG-D, TMEG, and ANME-1 groups. Variations in archaeal community composition reflect locally specific environmental challenges. Background sediments are divided into surface and subsurface niches. Overall, the environmental setting and history of a particular site, not isolated biogeochemical properties out of context, control the subseafloor archaeal communities in Guaymas Basin and Sonora Margin sediments.

## Highlights

• Archaeal community composition reflects locally specific environmental challenges
• Biogeochemical properties do not predict archaeal community structure
• Environmental history controls subseafloor archaeal populations

## Graphical Abstract

**Figure:**
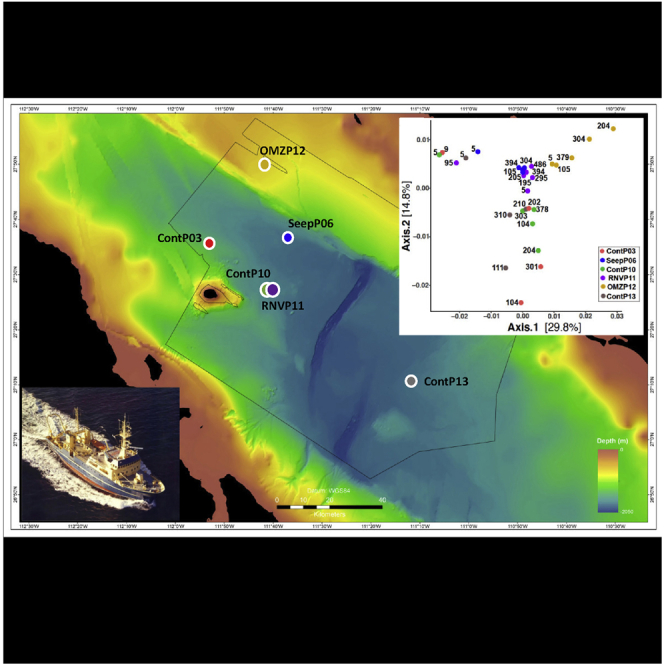

## Introduction

Guaymas Basin, located in the Gulf of California, México, is a young marginal rift basin where active seafloor spreading generates northeast-to-southwest trending axial troughs surrounded on both sides by extensive flanking regions (Lizarralde et al., 2007). In contrast to mid-ocean spreading centers, axial troughs and flanking regions of Guaymas Basin are covered by thick, organic-rich sediments that represent a combination of terrigenous input and biogenic sedimentation from the highly productive water column (Calvert, 1966). Magmatic intrusions, or sills, are embedded within the thick sediment layers, where they drive hydrothermal circulation (Lonsdale and Becker, 1985) and thermally alter buried organic matter (Seewald et al., 1990), in the process generating complex petroleum compounds (Didyk and Simoneit, 1989), light hydrocarbons and methane (Welhan and Lupton, 1987), carboxylic acids (Martens, 1990), and ammonia (Von Damm et al., 1985). Since the sediments act as a heat-retaining thermal blanket, magmatic activity and organic matter alteration and mobilization are not only limited to the spreading center but also occur at considerable distance, up to 50 km off-axis (Lizarralde et al., 2010). Many of these off-axis sites resemble cold seeps, where methane advection is linked to pathways formed by deeply buried magmatic sills (Geilert et al., 2018). If the underlying sill is sufficiently shallow and hot, the hydrothermal underpinnings of these off-axis sites becomes visible; the recently described Ringvent site provides an example (Teske et al., 2019).

In contrast, the Sonora Margin harbors classical cold seeps where sediment compaction drives reducing, methane-rich seep fluids to the surface. Numerous seep sites with carbonate outcrops and cold seep fauna have been observed on an eroding pressure ridge that follows the transform fault at the base of the Sonora Margin (Simoneit et al., 1990; Paull et al., 2007); the seep communities at these sites are largely based on methanotrophy and sulfide oxidation (Portail et al., 2015). Seep communities and sulfide-oxidizing microbial mats are also widespread on the Sonora Margin slopes (Vigneron et al., 2014; Cruaud et al., 2017).

Finally, most of the extensive flanking regions of Guaymas Basin and the Sonora Margin slope are covered by organic-rich sediments without particular seep or hydrothermal influence; these sediments consist of mixed terrigenous runoff and biogenic components, dominated by diatoms (Calvert, 1966). Sediments on the upper Sonora Margin underlying the oxygen minimum at ca. 600–800 m depth, lack bioturbation and show finely laminated, seasonally changing sedimentation patterns of spring diatom blooms and terrestrial runoff during late summer trains (Calvert, 1964).

Here, to explore links between geochemical and biogeographical patterns in geologically complex settings in the subseafloor, we survey the distribution of archaea in diverse sedimentary environments located in the greater Northern Guaymas Basin and Sonora Margin regions. Our samples were collected during a site survey with RV *El Puma* in October 2014 for the recently completed Integrated Ocean Discovery Program Expedition 385 (http://publications.iodp.org/scientific_prospectus/385/index.html). Sampling areas include background sediments from the Guaymas Basin flanking regions, Sonora Margin sediment within the oxygen minimum zone, reducing sediment with cold seep characteristics from the base of the Sonora Margin, and sediment from the off-axis Ringvent site where hydrothermal circulation and methane seepage is driven by a gradually cooling, buried shallow sill. We expand a previous limited sequencing survey of these sediments focused on just one of these sites (Teske et al., 2019) by (1) extending the geochemical analyses, (2) increasing the sampling resolution used for molecular sequencing (from one or two samples per site to ~1-m intervals for all sites), (3) providing a wide breadth of comparative ecological analyses, and (4) discussing the potential implications of our results at a basin-wide scale.

## Results

### Sediment and Porewater Geochemistry

**Figure 1:**
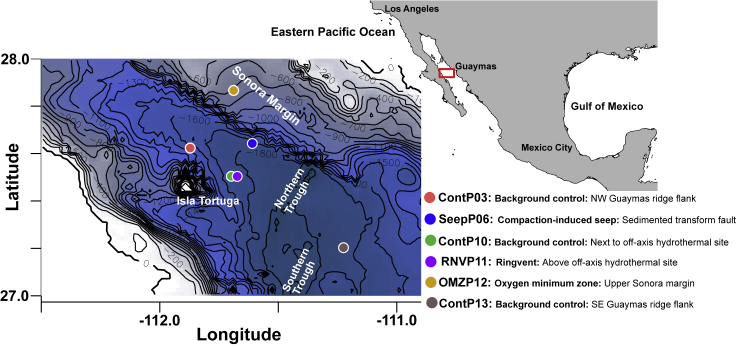
Continental and Bathymetric Hybrid Map Depicting the Location of Guaymas Basin and Sonora Margin in the Gulf of California, and Relevant Coring Sites of the *El Puma* Cruise The bathymetry blue scale is annotated with 100-m isobaths; the deepest areas in the axial valley range to just below 2,000 m.

**Figure 2:**
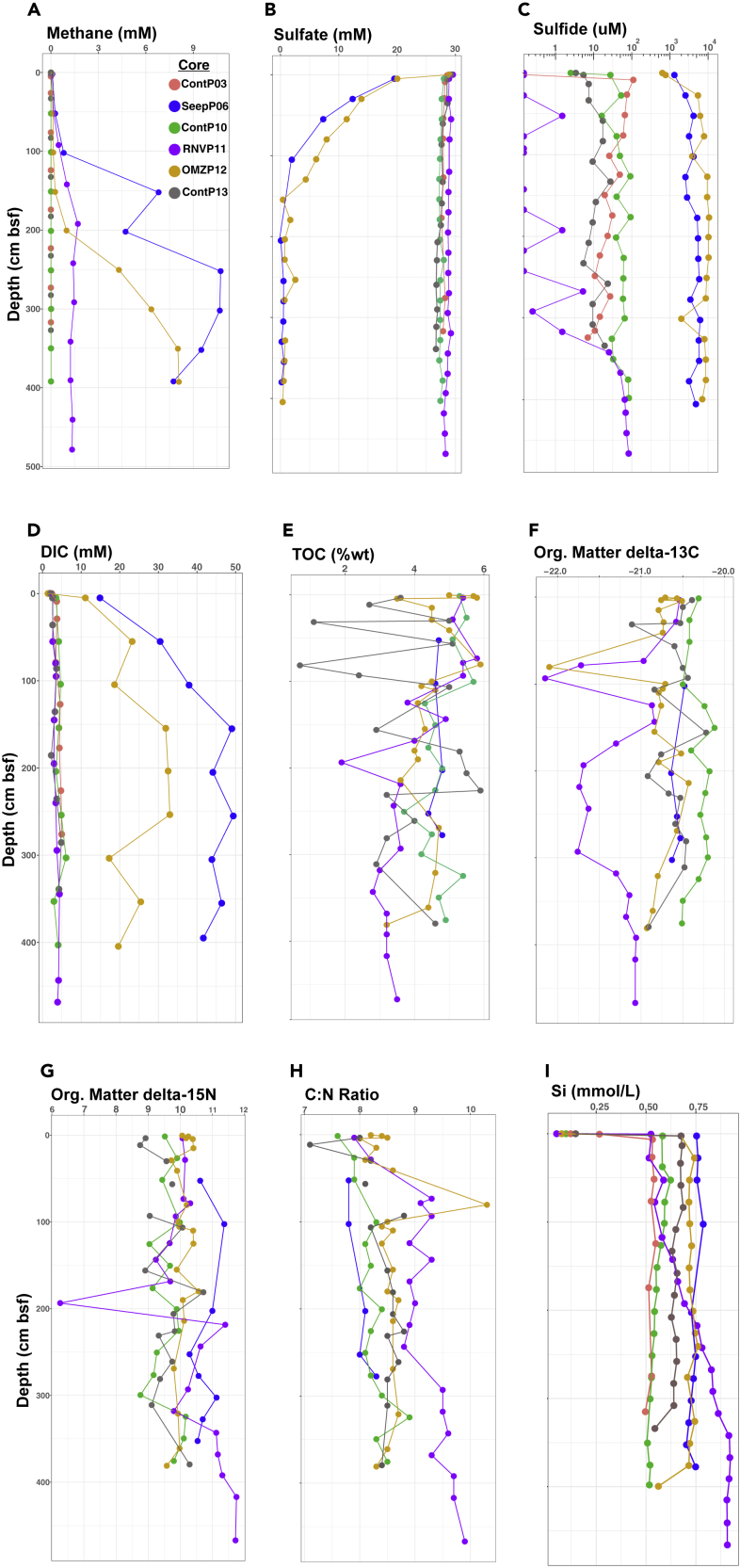
Geochemical Profiles for Guaymas Basin Piston Cores Geochemical profiles for (A) Methane, (B) Sulfate, (C) Sulfide, and (D) DIC porewater concentrations; (E) Organic Carbon content in weight %; (F) Organic Matter δ^13^C values, and (G) δ^15^N values; (H) Carbon to Nitrogen ratios, and (I) Silica porewater concentrations. Geochemical data for site ContP03 are not available for the analyses depicted in (E)–(H).

We surveyed archaeal distribution at six sites on the northwestern and southeastern off-axis regions of Guaymas Basin and on the Upper Sonora Margin (Figure 1, Table S1). These locations represent four different environmental settings: (1) Sediments on the Guaymas Basin flanking regions without hydrothermal or seep activity, represented by cores ContP03, ContP10, and ContP13; (2) the oxygen minimum zone (OMZ) on the upper Sonora Margin (Calvert, 1964), represented by core OMZP12, (3) compacted, highly reducing seep sediments from a pressure ridge, running along the transform fault that is cutting across the base of the Sonora Margin (Paull et al., 2007; Simoneit et al., 1990), represented by core SeepP06, and (4) the Ringvent site, characterized by off-axis hydrothermal circulation (Teske et al., 2019), represented by core RNVP11 (Figure 1). At each site, sediment piston cores ranging from 5 to 486 cm below the seafloor (cmbsf) were collected and geochemically characterized (Figure 2). The sediments are geologically young, ranging in age between ~0.05K and ~20K calendar years, as determined by ^14^C dating (Teske et al., 2019). The different cores show distinct geochemical characteristics.

Core SeepP06 contains sulfide in millimolar concentrations throughout the core. Below the zone of sulfate-dependent methane oxidation at 1 m depth, methane accumulated to the highest concentrations of this survey, > 10 mM. Porewater Dissolved Inorganic Carbon (DIC) concentrations were consistently high and increased from 15 mM near the interface to nearly 50 mM with depth. These methane and DIC concentrations reached and in part exceeded the highest concentrations previously measured in Sonora Margin seep fluids (Paull et al., 2007). Sediments of core SeepP06 yielded only approximately half of the porewater volumes of other cores, indicating porewater loss by pressure-induced compaction. Thus, core SeepP06 represents sulfidic, methane- and DIC-soaked, compacted seep sediments from the pressure ridge aligned with the transform fault at the lower Sonora Margin (Simoneit et al., 1990; Paull et al., 2007). Based on diatom assemblages in cemented carbonate rocks, seepage in this area has been ongoing since pre-Holocene times (Paull et al., 2007).

Cores ContP03 and ContP10 share similar methane, sulfate, sulfide, and DIC profiles indicative of non-reducing conditions where sulfate-reducing and methanogenic activities remain minimal and biogenic sulfide and methane occur only in micromolar trace concentrations. With total organic carbon (TOC) between 4 and 6 wt %, the sediments of core ContP10 are organic rich and represent the hemipelagic seafloor sediments of Guaymas Basin that receive ample biogenic sedimentation, mostly by diatoms (Calvert, 1966). The δ^13^C values ranging from −20.12‰ to −20.51‰ are consistent with sedimentary organic material resulting predominantly from phytoplankton input (Teske et al., 2002). Slowly increasing δ^15^N values ranging from 9.04‰ to 10.17‰ and gradually increasing C:N ratios downcore are consistent with microbial utilization of nitrogen compounds in sedimentary biomass.

Contrasting with nearby core ContP10, Core RNVP11 shows the biogeochemical signatures of seawater inmixing at Ringvent, such as seawater sulfate concentrations, and previous hydrothermal alteration, as evidenced by silica dissolution and re-precipitation (Teske et al., 2019). Subsurface-derived porewater methane in high concentrations of 1–1.5 mM coexists with porewater sulfate near seawater levels; sulfide is largely absent and reaches 10–100 μM only below 3 m depth. Core RNVP11 also stands out by having the lowest DIC concentrations of all cores, approaching seawater DIC in the upper layers. Below 1 mbsf, organic carbon δ^13^C values are the lowest for all cores (δ^13^C mean of −21.3), whereas C/N ratios are the highest (C/N mean ratio of 9.31), suggesting the influence of isotopically light and nitrogen-depleted hydrocarbons (Peter et al., 1991) (Figure 2). In contrast to other cores, Core RNVP11 shows a strong gradient of dissolved silica, increasing from 0.5–0.6 mM at the surface (similar to ContP10) to 0.9–1 mM at the bottom (Figure 2I). Silica dissolution requires a temperature window of 100°C–150°C and is therefore considered a marker of hydrothermal activity (Peter and Scott, 1988), ultimately resulting in elevated concentrations of dissolved silica in the water column of Guaymas Basin (Campbell and Gieskes, 1984).

Core OMZP12 differs from all other cores by its location in the oxygen minimum zone on the Sonora Margin slope (Calvert, 1964). Throughout its length, the core showed the conspicuous lamination that is typical for the absence of burrowing infauna and bioturbation in anoxic or severely hypoxic environments. Under these conditions, millimolar concentrations of porewater sulfide permeate the entire sediment core including the surface, otherwise only seen in compaction-induced seep sediments collected at the base of the Sonora Margin (core SeepP06). The sulfate-methane transition zone occurs at approximately 1 and 2 m depth for SeepP06 and OMZP12, respectively. Similar to core SeepP06, porewater DIC increases rapidly with depth, with a maximum value of 33 mM at 254 cmbsf. Sediment TOC, δ^13^C, δ^15^N, and C:N ratio values generally resemble those of other cores in this survey.

Core ContP13, collected on the southeastern flanking region, differs from other cores by terrestrial input from the Yaqui River. Methane, sulfate, sulfide, and DIC concentrations for this core follow similar depth profiles as observed for cores ContP03 and ContP10. However, TOC varies between ~1 and ~5 wt % in the first meter of sediment and between ~3 and ~6 wt % below, suggesting sedimentation pulses of varying organic carbon load. Sediment organic matter δ^13^C, δ^15^N, and C:N ratios fall within the range of values observed for other cores in this survey.

### Diversity of the Guaymas Basin Archaeome

**Figure 3:**
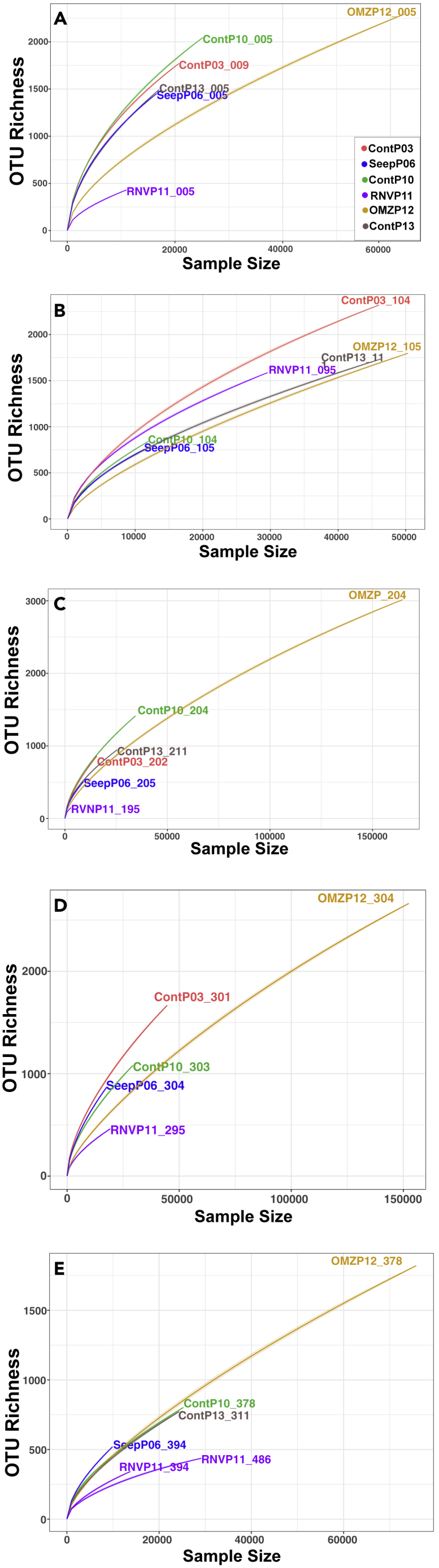
Depth Mapped Rarefaction Summaries (Color Coded to Match Surveyed Sites) for Complete High-Quality Sequence Datasets Depicting Richness as Number of OTUs (97% Similarity Clustered) Observed per Sequences Sampled (A) Samples near the interface (0–10 cmbsf), (B) samples from ~100 cmbsf, (C) samples from ~200 cmbsf, (D) samples from ~300 cmbsf, and (E) samples from depths greater than or approximately equal 400 cmbsf.

Rarefaction curves are plotted separately for samples in approximately 1-m depth intervals to examine potential downcore trends (Figure 3). Starting at 3 m depth, observed species richness based on rarefaction summaries are lower in Ringvent (Core RNVP11) sediment compared with other sediments (Figure 3, Table S2). Substantially more sequence reads, and thus a higher number of observed species, were recovered from the Sonora Margin OMZ sediment (Core OMZP12) relative to the other surveyed sites, below 1 m depth (Figures 3C–3E). To account for different sequencing depths without resorting to rarefying the dataset (McMurdie and Holmes, 2014), we estimated total diversity using a non-linear regression model for ratios of consecutive frequency counts, a state-of-the-art method addressing the issue of heterologous sequencing depths affecting richness estimates (Willis and Bunge, 2015). Results from this model indicate no statistically significant (P_val_ < 0.05) differences among surveyed sites (Figure S1).

**Figure 4:**
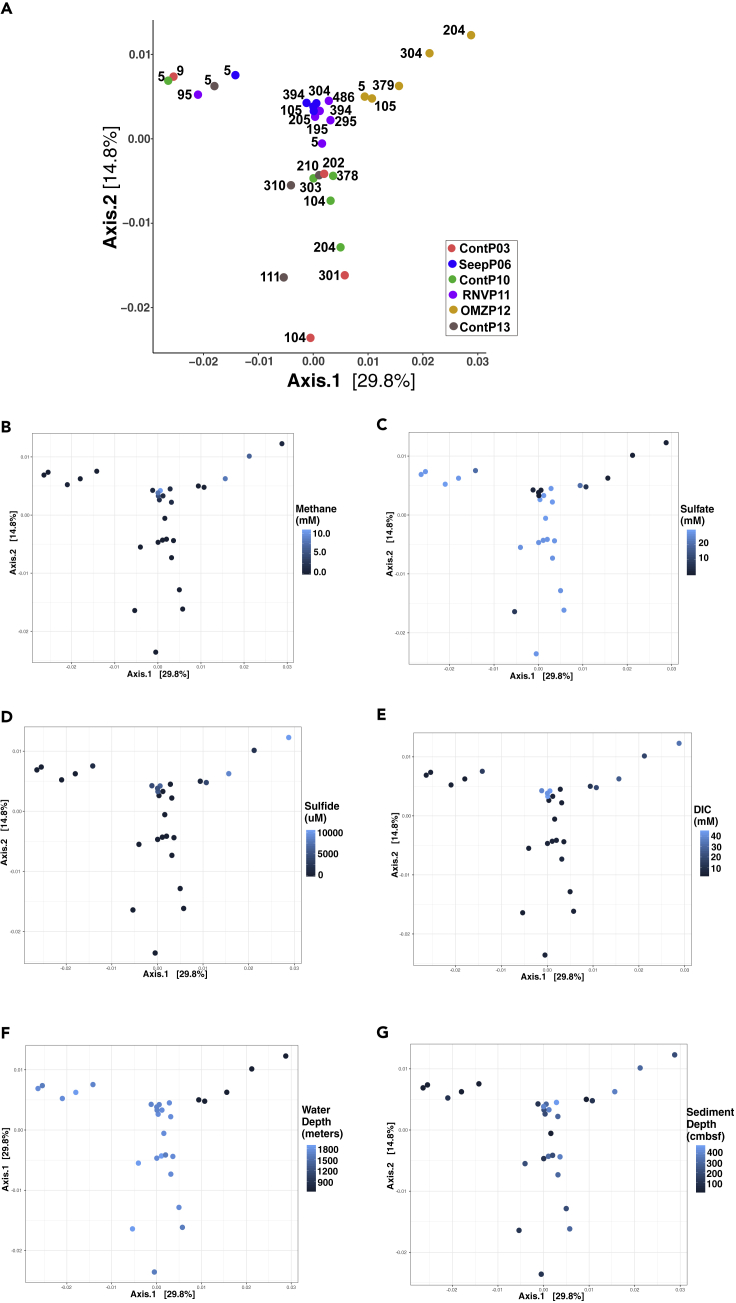
Archaeal Community Grouping Patterns (A) Two-dimensional Principal Coordinate Analyses of Bray-Curtis dissimilarity distances from r-log normalized sequence count data. Each community plotted is color coded to the core site and numerical labels indicate sediment depth (cmbsf). The first and second axes explain 29.8% and 14.8% of the variance, respectively. Environmental metadata superimposed on ordination plot are (B) methane, (C) sulfate, (D) sulfide, and (E) DIC concentrations, (F) water depth, and (G) sediment depth.

When beta diversity of the archaeal communities was examined for correlations with environmental metadata using two-dimensional principal coordinate analysis, distinct clustering patterns are observed (Figure 4A). Surface communities for all cores except OMZP12 cluster tightly along negative axis 1 values. All OMZP12 samples cluster along axis 1 values greater than 0.01 independently of sediment depth. Separation along axis 2 partitions the SeepP06, RNVP11, and OMZP12 cores (with positive axis 2 values) from subsurface samples of control cores ContP03, ContP10, and ContP13 with negative axis 2 values; the surface samples of these cores cluster separately (Figure 4A). The influence of environmental factors (i.e., methane, sulfate, sulfide, DIC, water depth, and sediment depth) on community ordination is complex (Figures 4B–4G), and it appears likely that clustering patterns are not driven by these environmental parameters alone. Water column depth (Figure 4F) appears to drive core OMZP12 clustering along larger positive values for axis 1 but most likely represents a proxy for the influence of the OMZ that has persisted throughout the Holocene (Moffitt et al., 2015).

### Network Analysis

**Figure 5:**
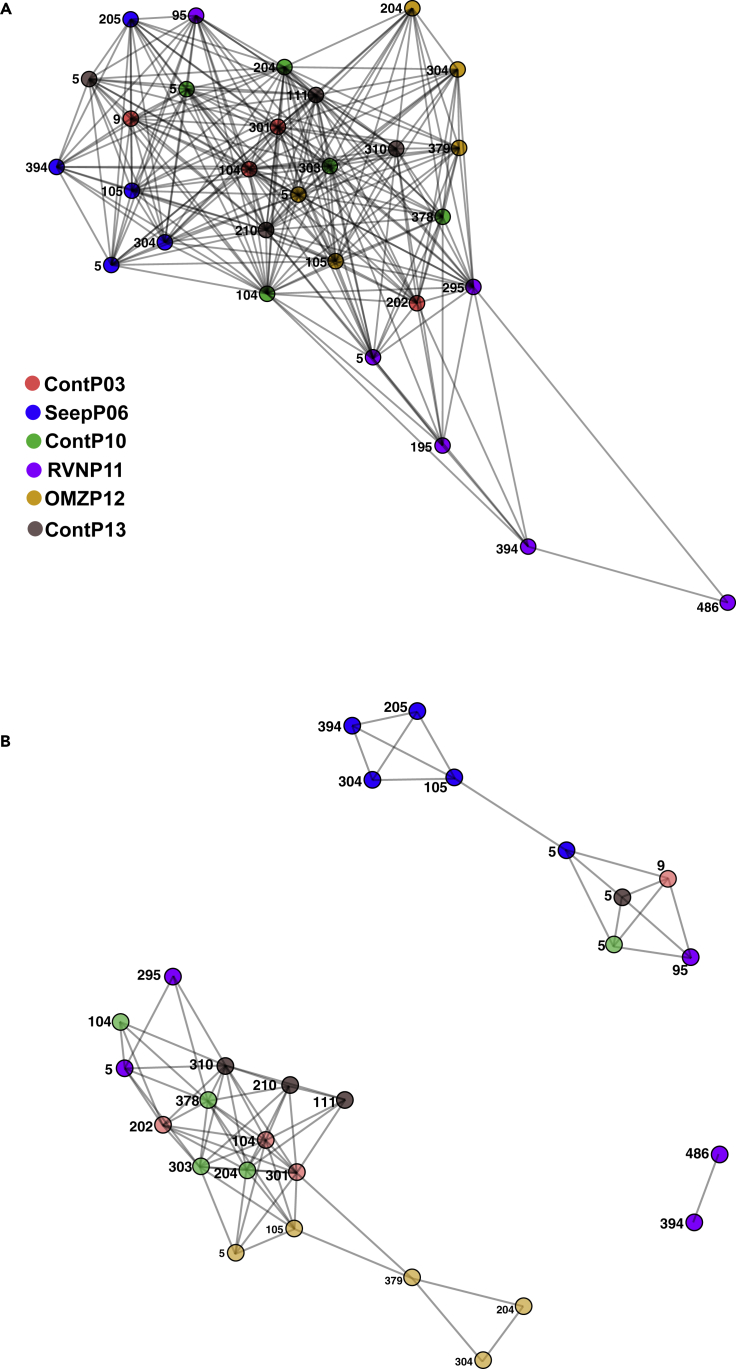
Network Analysis Based on the Co-occurrence of All OTUs at in Each Sample Nodes represent all archaeal communities analyzed in this study. Nodes are color coded to match descriptions from Figure 1A. Edges are unweighted interactions depicting OTU co-occurrence meeting arbitrary thresholds. (A) Co-occurrence network threshold set at a maximum Bray-Curtis distance of 0.8. (B) Co-occurrence network threshold set at a maximum Bray-Curtis distance of 0.5.

Network analysis based on the co-occurrence of all operational taxonomic units (OTUs) in each sample reveals that the deepest communities recovered from Core RNVP11 exhibit the greatest degree of separation (Figure 5). At a maximum ecological (Bray-Curtis) distance of 0.8 (i.e., the maximum distance allowed between two samples to be considered connected in the graphical model), most samples share taxa co-occurrence patterns, except for the deepest communities from core RNVP11 (Figure 5A). Decreasing the minimum ecological distance in the model to 0.5 resolves three independent network clusters (Figure 5B). Here, the two deepest samples from core RNVP11 share similar taxa co-occurrence patterns only with each other and are excluded from the two emergent additional networks. In one of these networks, communities near the seawater interface of all cores, with the exception of core RNVP11, connect at no more than 1 degree of separation. Interface sample SeepP06 5cmbsf connects the near-interface sample cluster to all core SeepP06 subseafloor (depth > 1 mbsf) samples. A third independent network shows non-random taxa co-occurrence among subseafloor control sediments (cores ContP03, ContP10, ContP13), a subseafloor and a near-interface sample from core RNVP11, and all samples from core OMZP12; the three deepest OMZP12 samples are only peripherally connected (Figure 5B).

### Community Composition

**Figure 6:**
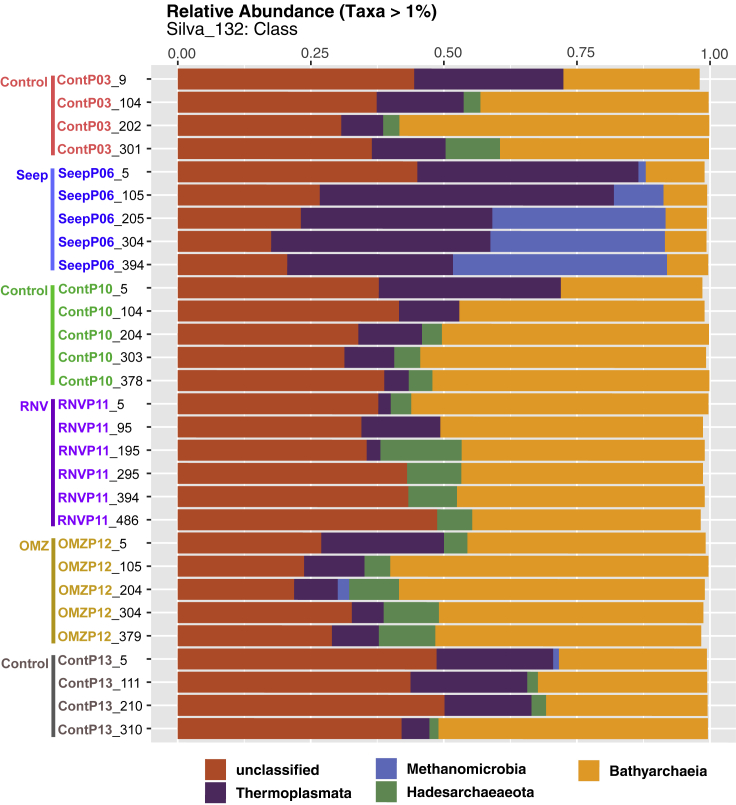
Class-Level Community Composition of All Depths for All Cores in This Study Core labels are color coded to match the collection sites depicted in the bathymetric map in Figure 1.

Class-level community descriptions (SILVA132) assigned large membership fractions of the archaeal communities to the Bathyarchaeia, Hadesarchaeaeota, and Thermoplasmata (Figure 6). Asgardarchaea (i.e., sequences classified as Lokiarchaea, specifically) were recovered from every cored site; however, their percent community abundance remained below 0.01% in all samples (Table S2). We note that, when a subset of these samples was studied using a different Archaeal-specific primer set, higher percentages of Lokiarchaea were observed (Teske et al., 2019), implying a potential bias against this lineage in this study. The Class Methanomicrobia, comprising methane-producing and methane-oxidizing members of the Euryarchaeota, was detected in multiple cores but appeared most frequently at depth in core SeepP06. An in-depth summary of the Methanomicrobia reveals the presence of methanogenic families (e.g., Methanomicrobiaceae) and anaerobic, methane-oxidizing ANME lineages (ANME-1, various ANME-2). Notably, ANME-1 archaea dominate core SeepP06 sequence assignments comprising nearly 40% of the total community at 394 cmbsf in this core (Figure S2, Table S3). Order- or higher-level community taxonomic descriptions for all samples generally contained 60% or greater unclassified community fractions (data not shown) when automated taxonomic assignments were performed. In order to not rely on the uncertain output of taxonomy pipelines, and to resolve archaeal taxonomy assignments in a manner that is consistent with broadly accepted usage (Spang et al., 2017), we also describe community composition based on phylogenetic placement of dominant sequence variants for the most numerous 25 OTUs.

**Figure 7:**
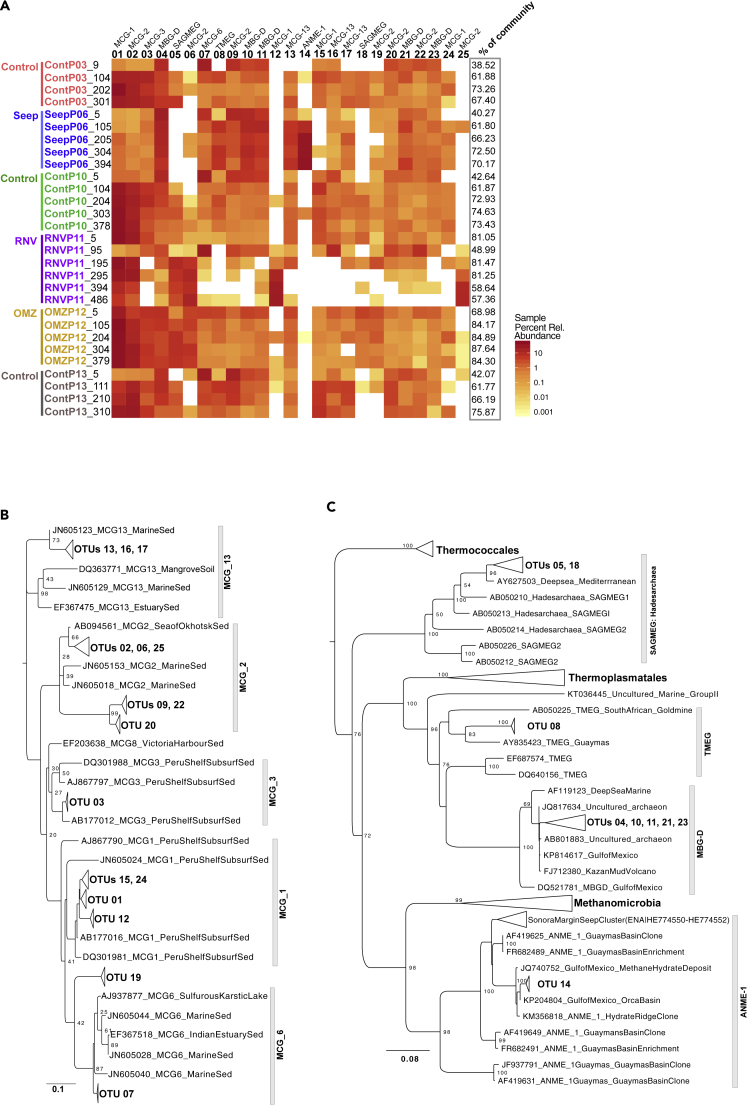
Distribution and Phylogeny of Dominant Archaeal OTUs (A) Heatmap depicting percent relative abundance distribution for the 25 most abundant OTUs, representing 73.0% of all high-quality sequences in this study, for all cores and across all depths. Core labels are color coded to match the collection sites depicted in the bathymetric map in Figure 1A. The phylogenetic association of each OTU lineage is depicted above each OTU header. The percent of total reads represented by the 25 most abundant OTUs in each community is shown in the column labeled “% of community.” Maximum likelihood phylogenetic trees, with 100 bootstrap support, placing the top 25 most abundant OTUs within the following lineages: (B) Bathyarchaea, (C) the Euryarchaeotal lineages MBG-D, TMEG, SAGMEG, and ANMEs.

The majority of high-quality sequences in this study (73.0%) clustered into 25 OTU lineages (Figure 7A). Archaeal communities were largely dominated by OTU lineages related to the Bathyarchaea (16 of the top 25 OTUs). OTUs 01 to 03, the three most abundant lineages, belong to the MCG-1, MCG-2, and MCG-3 Bathyarchaea subgroups (Kubo et al., 2012), respectively, with close relatives recovered from Guaymas Basin and globally dispersed subseafloor habitats (Figure 7B). High-abundance lineages related to the Marine Benthic Group-D within the Thermoplasmata (MBG-D; OTUs 04, 10, 11, 21, and 23) and the Terrestrial Miscellaneous Euryarchaea Group (TMEG; OTU 08) were recovered from all cores and core depths except the subsurface of Ringvent (Core RNVP11, depth > 1 mbsf). Two highly abundant lineages represented by OTUs 05 and 18 (Figure 7C) were recovered from every core except core SeepP06 and identified as relatives of the Hadesarchaea, formerly known as South African Gold Mine Euryarchaea Group (SAGMEG)-1 (Baker et al., 2016). Lastly, OTU 14 clustered within anaerobic ANME-1 methanotrophs and was most closely related to ANME-1 phylotypes from cold seep, hydrate, and brine habitats; OTU 14 did not affiliate with the thermophilic ANME-1 Guaymas lineage recovered from hydrothermally active, hot sediments in Guaymas Basin (Biddle et al., 2012) (Figure 7C).

### Differential Taxon Abundance Estimations across Ecological Niches

**Figure 8:**
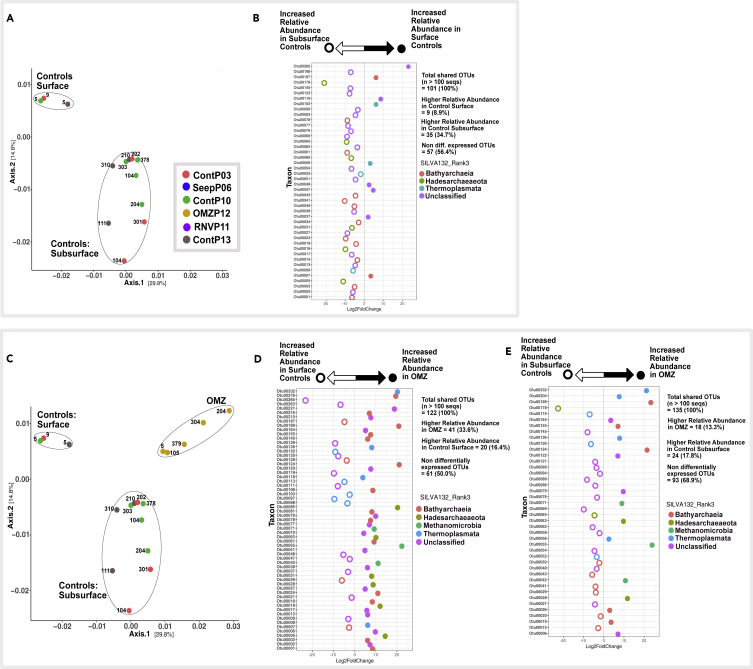
Differential Abundance Analyses Based on Wald's Test (Significance: alpha = 0.01) (A) Ordination depicting archaeal community clustering for surface and subsurface samples of control sites ContP03, ContP10, and ContP13. (B) Differentially abundant OTUs in near-surface versus subsurface communities from control sites. (C) Ordination depicting community clustering in OMZP12, and surficial versus subseafloor control sites. (D and E) (D) Differentially abundant OTUs in OMZP12 compared with surficial and (E) subsurface communities from control sites. Note: OTUs 13, 17, and 21 are color coded as “unclassified” by SILVA132, but phylogeny analysis (see Figures 7B and 7D) places them as members of MCG-13 (OTUs 13 and 17) and MBG-D (OTU 21).

**Figure 9:**
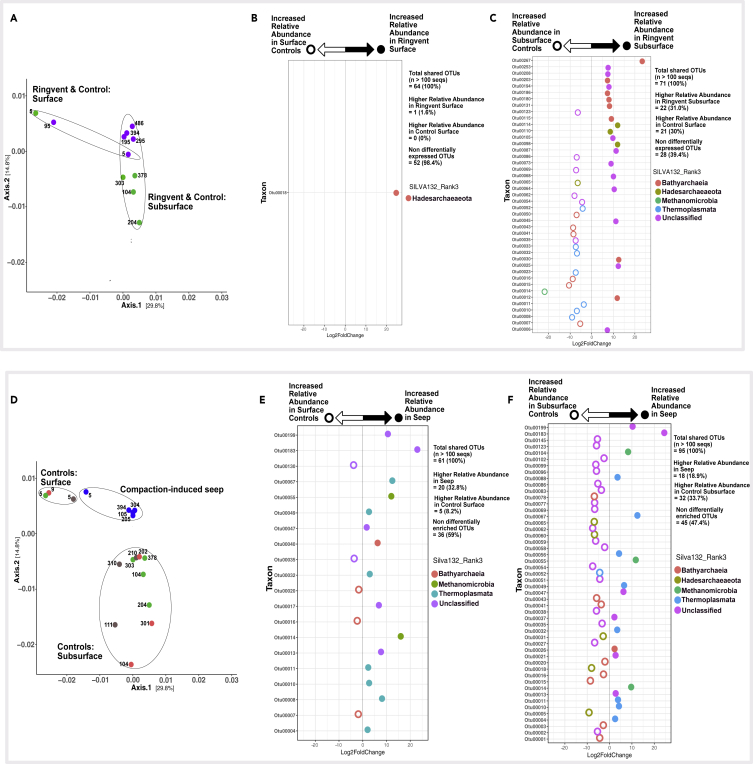
Differential Abundance Analyses Based on Wald's Test (Significance: alpha = 0.01) (A–C) (A) Ordination depicting community clustering in RNVP11 and ContP10. Differentially abundant OTUs in RNVP11 and ContP10 for (B) surface samples and (C) subsurface samples. (D) Ordination depicting community clustering in SeepP06, and surficial and subseafloor control sites. (E and F) Differentially abundant OTUs in SeepP06 samples compared with (E) surficial and (F) subsurface communities from control sites. Note: OTUs 13, 17, and 21 are color coded as “unclassified” by SILVA132, but phylogeny analysis (see Figures 7B and 7D) places them as members of MCG-13 (OTUs 13 and 17) and MBG-D (OTU 21).

Differential abundance analyses (Wald Test, Pval = 0.01) were performed on various ecological models following potential environmental niches suggested by ordination patterns (Figures 8 and 9). Only OTU lineages among comparison groups containing more than 100 sequences were used for each test. We tested the influence of sediment depth in the absence of seepage or hydrothermal influence, the impact of the oxygen minimum zone waters on surficial and subsurface sediments, and the effect of hydrothermal disturbance. These analyses have to be qualified by the fact that they are based on patterns of sequence frequencies, which are derived from the archaeal community but do not necessarily represent it in identical proportions.

To test the estimated differential abundance of archaeal community members in near-surface (depth < 1 mbsf) and subsurface (depth > 1 mbsf) communities under conditions of normal hemipelagic sedimentation, we selected cores ContP03, ContP10, and ContP13; these cores lack hydrothermal, seepage, or OMZ influence and, therefore, show the archaeal community of organic-rich Guaymas Basin sediments in the absence of these selective factors. Here, over 43% of OTUs (n > 100 seqs) are differentially abundant with depth (Figures 8A and 8B). Most differentially abundant taxa are estimated to have lower relative abundances in near-surface relative to subsurface control sediment (Figure 8B). Lineages with higher subsurface relative abundances include members of various Bathyarchaeal groups (MCG-1, MCG-3, and MCG-6), a TMEG lineage (OTU 08) closely related to clones previously recovered from Guaymas Basin, and a Hadesarchaea lineage (OTU 18) whose closest relatives are clones from deep Mediterranean waters (Figures 7B and 7C). Among the top 25 OTUs in this study, the only highly abundant lineage that significantly (Pval = 0.01) increases in relative abundance in near-surface relative to subsurface control sediment (Figure 8A) is OTU 07, related to Guaymas Basin and Indian estuary sediment MCG-6 clones (Figure 7B). The same lineage was also found to occur preferentially in surficial estuarine sediments in the White Oak River, while avoiding the sulfide-rich, sulfate-reducing, and methanogenic conditions just a few centimeters downcore (Lazar et al., 2015).

When archaeal abundance in all oxygen minimum core OMZP12 samples was checked against the shallow sediment samples of the control cores (ContP03, ContP10, ContP13, see Figures 8C and 8D), over 50% of archaeal OTUs (n > 100 seqs) present in surficial controls showed higher relative abundances in core OMZP12. Of the 25 most highly abundant OTUs in this study, those that were differentially abundant in this comparison (14 OTUs) increased in relative abundance in core OMZP12 (eleven of fourteen high abundance OTUs, Figure 8D). Only OTUs 07, 09, and 21, representing the Bathyarchaeal groups MGC-6 and MCG-2, and an MBG-D lineage, respectively, were more abundant in surficial control sediment (Figure 8D).

We also tested for differentially abundant taxa between all core OMZP12 samples and subsurface control sediment (Figure 8E). Here, only 31.1% of OTUs (n > 100 seqs) were differentially abundant; of the 25 most abundant OTUs in this study only 4 showed significant abundance differences. Of these four archaeal OTUs, OTUs 15 and 20, Bathyarchaeal lineages in the MCG-1 and MCG-2, respectively, increase in relative abundance in subsurface control sediment. OTU 06 within the MCG-2 group, and OTU 19, an MCG lineage tenuously related (bootstrap value <70%) to MCG-6, decreased in relative abundance in core OMZP12. Overall, archaeal types occurring in anoxic subsurface sediments of core OMZP12 resemble those in other subsurface cores to a large extent.

The hydrothermally influenced Ringvent core RNVP11 was compared against control core ContP10, located only 1.6 km further west, at the same depth and local sedimentation regime (Figures 9A–9C). In surficial sediment (>1 mbsf) this comparison revealed only one differentially abundant phylotype, OTU 18, within the Hadesarchaea (Figure 9B). On the other hand, the comparison of subsurface (>1 mbsf) communities identified 43 OTUs, or 61% of all shared OTUs (n > 100 seqs), as differentially abundant between these two sites (Figure 9C). Focusing on the top 25 highest abundance OTUs in this study, eleven OTUs were differentially abundant. Eight taxa, comprising Bathyarchaeal, TMEG, MBG-D, and ANME-1 representatives, were significantly more abundant in subsurface control sediment relative to Ringvent subsurface sediment (OTUs: 07, 08, 10, 11, 14, 15, 16, and 23). The remaining three taxa (OTUs 06, 12, and 25), more abundant in the Ringvent subsurface, were classified as Bathyarchaea related to the MCG-1 and MCG-2 subgroups.

Core SeepP06, from compacted seep sediments, was checked against both shallow and subsurface sediment samples of the control cores (ContP03, ContP10, ContP13, Figures 9D–9F). About 41% of archaeal OTUs (n > 100 seqs) present in shallow controls and seep sediment were significantly differentially abundant (Figure 9E). Of the 25 most highly abundant OTUs, most showed higher relative abundances in seep sediment and included lineages classified as ANME-1, MBG-D, and TMEG (Figure 9E). When comparing differentially abundant taxa between core SeepP06 and subsurface control sediment, 53% of archaeal OTUs (n > 100 seqs) were differentially abundant (Figure 9F). Most of the OTUs in this comparison, including the most abundant OTUs in this study (OTUs 01–03), increase in relative abundance in control subseafloor sediments rather than in core SeepP06 sediment (Figure 9F).

## Discussion

### Complex Determinants of Archaeal Ecosystem Structure

Overall, complex physical and geochemical factors structure sedimentary habitats and depth-related niches for archaea in Guaymas Basin. Archaeal community ordination patterns reveal niche differentiation and some unexpected clustering patterns among the different sites (Figure 4A). Most notably, surficial communities of background control sediments ContP03, ContP10, and ContP13, at 5–10 cm depth, cluster away from their respective subsurface communities near 1 m depth and below (Figure 4A), consistent with infaunal bioturbation and aeration as observed during Guaymas Basin *Alvin* dives. Taxa co-occurrence network patterns support this differentiation between shallow and subseafloor control sediment sites (Figure 5B). The availability of electron acceptors such as oxygen, nitrate, or oxidized metals very likely drives the depth-dependent niche separation observed in background control sediment sites (ContP03, ContP10, and ContP13). Surface archaeal communities continually change as sediment layers accumulate; given high sedimentation rates of 0.23–1 mm/year at Guaymas Basin (Teske et al., 2019), it takes approximately 1,000–4,000 years for background surface communities to transition to subsurface communities at 1 m depth. Interestingly, shallow versus subsurface differentiation is less apparent in seep, OMZ, or hydrothermally influenced sites (Figure 5B); parameters other than sediment depth or surficial redox regime are shaping archaeal community composition in seepage- or hydrothermally influenced habitats, compared with the control sites.

The cores SeepP06 and RNVP11 represent different geochemical regimes (compaction-induced continental margin seepage versus hydrothermal circulation, respectively), yet these two sites cluster tightly in ordination space (Figure 4). Individual geochemical factors, for example, the sulfidic, methane-, and DIC-rich conditions in SeepP06 would have indicated that OMZP12 should be its closest equivalent (Figure 2). The unexpected clustering of SeepP06 and RNVP11 suggests that factors beyond current geochemical conditions, for example, recent environmental disturbance, can influence archaeal community structure. For example, variable ^14^C age data for the sediment column in core SeepP06 suggested prior perturbation by slumping, for example, during steep slope collapse (Teske et al., 2019). At Ringvent (RNVP11), sedimentary community diversity may have been reduced during prior episodes of thermal purging or high methane flux (Teske et al., 2019), selecting for a resilient, yet potentially less diverse (Figure 3), “survivor” community.

Lastly, community ordination differentiates OMZ sediment from all other sedimentary habitats (Figure 4). Although water depth appears to have a strong influence on OMZ sediment ordination (Figure 4F), we propose that differences in redox potential at the sediment interface due to its direct contact with oxygen-depleted water (Calvert, 1964) rather than water column depth is the key environmental constraint driving the ordination patterns of OMZ sediment. A distinct archaeal community is consistent with the persistence of fully developed oxygen minimum conditions on the Sonora Margin since approximately 13,000 years (Moffitt et al., 2015).

### A “Forest View” of Archaea in Guaymas Basin Sediments

Archaea observed in this sedimentary habitat survey belong to the Bathyarchaea, the MBG-D and TMEG lineages within the Thermoplasmatales, the Hadesarchaea (SAGMEG), and ANME-1 lineages, as shown previously in a sequencing survey using different archaeal primers (Teske et al., 2019). The uncultured Bathyarchaea and MBG-D archaea pronouncedly dominate the dataset, and cold anoxic marine sediments globally (Kubo et al., 2012; Lloyd et al., 2013). Bathyarchaea play an important role, tantamount to that of the domain Bacteria, in the remineralization of complex organic matter in marine sediment (Lloyd et al., 2013; Orsi et al., 2020); some of their members (MCG-8 lineage) use lignin, the second most common biopolymer on Earth, as an energy source (Yu et al., 2018). Since Bathyarchaeota harbor the Wood-Ljungdahl pathway (WLP), they are implied in acetogenic subsurface metabolism (He et al., 2016). Recycling fermentation products (CO_2_, hydrogen) with the acetogenic WLP during the breakdown of diverse substrate types may provide the Bathyarchaea with an energetic advantage over classical fermenters in anoxic sediment (Schuchmann and Müller, 2016). It remains open whether acetogenic pathways are used for net autotrophy or derive their substrates primarily from organic carbon sources (Lever et al., 2010); similar considerations apply to the metabolically versatile MBG-D archaea (Zhou et al., 2019). Some Bathyarchaea harbor genes of the MCR complex, suggesting methylotrophic methanogenic activity and, perhaps, syntrophic interactions with sulfate-reducing bacteria leading to the anaerobic oxidation of methane (Evans et al., 2015). However, the dominant Bathyarchaea OTUs recovered in this study (MCG groups 1, 2, 3, 6, and 13, Figure 7B) are only distant relatives of methane-cycling marine Bathyarchaea, which fall into MCG groups 15 and 16 (Evans et al., 2015). Hadesarchaea, originally described as the South-African Gold Mine Miscellaneous Euryarchaeal Group (SAGMEG), are metabolically versatile anaerobic heterotrophs with the metabolic potential for CO and H_2_ oxidation coupled with nitrite reduction to ammonia and are found in environments across broad (4°C –80°C) thermal ranges (Baker et al., 2016). One of two frequently recovered Hadesarchaea lineages (OTU 05) is conspicuously present in high relative abundance in subsurface sediment in Ringvent (RNVP11), where low observed sequence richness (Figure 3) coincides with evidence (high silica porewater concentrations at depth indicative of a thermal dissolution of sedimentary diatoms, Figure 2I) for a thermal purge in the past (Teske et al., 2019).

Unsurprisingly, the methane-cycling Methanomicrobia are rare in background sediments (ContP03, Cont10, and Cont13) but increase in relative abundance in core SeepP06 and, to a much lower extent (slightly over 2%), in core OMZP12 at 204 cm depth (Figures 6 and S2). Interestingly, a single ANME-1 OTU lineage, OTU14, is highly abundant in SeepP06 sediments (Figures 7A and 7C). This lineage is closely related to ANMEs recovered from cold, anoxic habitats, such as seafloor seep sediments, methane hydrates, and hypersaline anoxic basins, and distinct from previously described ANME-1 phylotypes from Sonora Margin cold seep sediments and potentially thermophilic ANME-1 phylotypes from Guaymas Basin hydrothermal sediments (Biddle et al., 2012; Holler et al., 2011). Although ANME-1 archaea were generally assumed to be obligate methanotrophs, this assumption has been challenged and this lineage has been proposed as potentially methanogenic, based on its occurrence and activity in sulfate-depleted sediments (Lloyd et al., 2011; Kevorkian et al., 2020); thus, the biogeochemical role of these archaea would be modulated by the presence or absence of sulfate or concomitant changes in electron donors. ANME-2 and cultured methanogenic lineages were observed in low percent abundances in all cores in this study (Figure S2). Interestingly, ANME-2 lineages were extremely rare, representing less than 0.05% of any sample and less than 0.02% of any SeepP06 community (Figure S2, Table S3). The prevalence of ANME-1 over ANME-2 in our survey is consistent with the ecophysiological preference of ANME-1 archaea for reducing, sulfidic subsurface sediments and the preference of ANME-2 for near-surface sediments with intermittently oxidizing conditions (Ruff et al., 2015; Rossel et al., 2011). Previous surveys of mat-covered seep sediments on the Sonora Margin have revealed transitions from ANME-2 toward ANME-1 within short push cores of maximum 17 cm depth (Vigneron et al., 2013).

Overall, we hypothesize that the archaeome in the sedimented flanking regions of Guaymas Basin is generally fueled by heterotrophic processes including the degradation of proteins, polymeric carbohydrates (Ziervogel and Arnosti, 2020), and accumulating lipids (Teske et al., 2002) resulting from high sedimentation rates driven by high levels of primary production in the water column. Diverse niche communities allow the Guaymas archaeome to adapt to environmental challenges, such as hydrothermalism or methane seepage, that are common in the greater Guaymas Basin area.

### Ecological Comparisons: Differentially Abundant Taxa across Sedimentary Habitats

#### Near-Surface versus Subsurface Sediment Niches

When comparing the surficial to the subsurface archaeal populations in background control sediments, the majority of OTUs estimated to be differentially abundant are significantly more abundant in the subsurface relative to the surficial sediment, suggesting that benthic archaea prefer subsurface conditions (Figures 8A and 8B). This trend may also reflect the impact of electron acceptors; for example, oxygen permeates background sediments in Guaymas Basin for at least 1 cm (Teske et al., 2016, Figure 8B therein). Following a recently proposed model for benthic microbial communities (Starnawski et al., 2017) the archaeal community at the oxic water-sediment interface likely undergoes downcore selection, based on site-specific selective pressure, resulting in reduced diversity with depth but a higher prevalence of subsurface-adapted taxa within a few thousand years after burial in anoxic subseafloor sediment. Benthic archaea, predominantly Bathyarchaea and MBG-D lineages, survive on residual carbon sources that remain after burial and microbial degradation in surficial sediments (Lloyd et al., 2013). Interestingly, catabolic activity and electron donor diversity, rather than terminal electron acceptor type or burial time, appear to drive bacterial OTU richness in anoxic subseafloor sediment (Walsh et al., 2016). This niche construction mechanism, driven by the biotic microenvironment as opposed to abiotic environmental filtering (Aguilar-Trigueros et al., 2017), is potentially widespread across the large habitable volume represented by non-hydrothermally influenced subseafloor sediments in Guaymas Basin.

#### OMZ versus Control Sediment

When comparing OMZ and surficial background control sedimentary communities, 50% of high abundance OTUs found across both habitats are significantly differentially abundant (Figure 8D). Two-thirds of the differentially abundant taxa have higher relative abundances in the OMZ rather than the surficial background sediments. The MCG lineages MGC-6 and MCG-2 (OTUs 9 and 7) and an MBG-D phylotype (OTU 21) increase in relative abundance in the surficial background controls relative to the OMZ sediment (Figure 8D). Interestingly, MCG-6 members bear hydrolases that specifically target plant-derived polymeric carbohydrates (Lazar et al., 2016), a potential trait-environment relationship that may differentiate surficial background control from OMZ sediment communities (Figure 8D). When comparing subsurface background control and OMZ sediment communities, the number of differentially abundant OTUs was about equal across both environments; however, the majority (68.9%) of high abundance OTUs show no significant differences in differential abundances across subsurface background controls and OMZ environments (Figure 8E). This implies that the subsurface, rather than surficial, background control communities are more similar to the OMZ communities, a point also corroborated by taxa co-occurrence network analysis (Figure 5B). Thus, oxygen depletion in background subsurface sediment and oxygen depletion through the overlying oxygen minimum zone of the water column (Calvert, 1964) result in some convergence between archaeal communities across geographically distant and environmentally distinct sedimentary habitats.

#### Ringvent versus Control Sediment

The surficial archaeal communities of Ringvent (RNVP11) and its nearby control site (ContP10) are similar to each other, as indicated by extensive co-occurrence networks (Figure 5) and by the dearth of differentially abundant OTUs between the two cores (Figure 9B). A member of the Hadesarchaea, OTU18, is the only differentially abundant lineage in Ringvent surficial sediment relative to the control; otherwise differences in taxon relative abundance across these habitats are negligible. These sites are only 1.6 km apart and therefore most likely share recent depositional histories and microbial inoculum sources, which validates core ContP10 as a site-specific control for assessing the environmental determinants structuring subsurface archaeal communities at Ringvent. The reduction in sequence recovery and, potentially, archaeal community richness in subsurface Ringvent (RNVP11) sediment (Figure 3) is attributed to environmental selection via hydrothermal purging as reflected in silica dissolution, or methane seepage driven by recent sill emplacement that continues to drive hydrothermal circulation, selecting against microbes unable to withstand these chemical or thermal changes (Teske et al., 2019). Thus, OTUs with increased relative abundances in Ringvent subsurface sediment compared with its nearby control site (Figure 9C) may occur via two possible ecological scenarios: (1) surviving resilient microbes could dominate the habitat after their competitors have been removed and (2) new arrivals after the disturbance could efficiently recolonize the depopulated surface sediment.

#### Seep versus Control Sediment

Differential abundance comparisons show that the ANME-1, MBG-D, and TMEG lineages increase in relative abundance in the seep sediments, compared with controls (Figures 9D and 9E). Generally, methane seeps are specialized microbial benthic habitats where methanotrophic archaea (ANME) and syntrophic Deltaproteobacteria oxidize methane anaerobically exploiting sulfate as an electron acceptor (Lloyd et al., 2010; Ruff et al., 2015). The dominance of these inter-domain syntrophic partners distinguishes seafloor seep habitats (Ruff et al., 2015). Archaeal community structure in SeepP06 sediments differs little with depth; it is most similar, in terms of taxa overlap, to other samples from the same core (Figures 4 and 5). Therefore, the influence of cold seepage drives community selection to a greater degree than the environmental factors associated with depth-dependent niche differentiation observed in background control sediment.

Comparison with other Sonora Margin cores highlights the seep characteristics of core SeepP06. Based on the presence or absence of major archaeal lineages, SeepP06 archaeal communities are similar to surficial (<1 mbsf) communities from Sonora Margin cold seeps, predominantly comprising Thermoplasmata (MBG-D), Bathyarchaea, and ANME lineages (Cruaud et al., 2017). The SeepP06 archaeal communities share dominant archaeal lineages—the Thermoplasmatales (MBG-D), Lokiarchaeota, and Bathyarchaeota—with Sonora Margin subsurface sediments (core BCK1 [Vigneron et al., 2014]). Interestingly, the high proportion of ANME-1 archaea in SeepP06 is not shared by the Sonora Margin subsurface core (Vigneron et al., 2014). The Sonora Margin subsurface sediment core has a deeper methane/sulfate interface than SeepP06, ca. 4–5 m instead of 1 m, and contains little sulfide above 5 m depth, indicating strongly attenuated seep influence in core BCK1 compared with SeepP06.

#### Core-Specific Features of the Benthic Archaeome

Controls that structure microbial communities in hydrothermal sediments of Guaymas Basin have been studied extensively; for example, extreme temperature and porewater gradients shape microbial population structure, genomic repertoire, and activities within a few centimeters depth beneath the seafloor (McKay et al., 2012, 2016; Dombrowski et al., 2018). However, ecological factors influencing microbial life in other sedimentary habitats at Guaymas Basin are comparatively unconstrained. By comparing archaeal communities in diverse sedimentary habitats to background controls representative of standard hemipelagic sedimentation, characteristic responses of the archaeal communities to these distinct environmental settings are becoming apparent. Compaction-induced seepage near the base of the Sonora Margin, and the resulting methane- and sulfide-rich porewater conditions in core SeepP06, selected for anaerobic methane-oxidizing archaea (ANME-1) and for MBG-D archaea within the Thermoplasmata, and reduced the relative proportion of Hadesarchaea and Bathyarchaeota. Prior disturbances by hydrothermal impact or strong methane seepage, exemplified in the Ringvent sediments (RNVP11), also strongly differentiated sedimentary archaeal communities from those in background controls. Observed community richness in RNVP11 based on rarefaction curves appears reduced throughout much of the core; these results resembled the outcome of a parallel study using different archaeal primers, and bacterial primers as well (Teske et al., 2019). Lastly, anoxic bottom waters impinging on the sediment on the upper Sonora Margin (OMZP12) drive similarities between anoxic surficial sediment at this site and anoxic subsurface background control sediments. The anoxic redox state of the water-sediment interface may also enhance archaeal richness estimates in the upper sediment column, potentially by facilitating the pelagic-benthic transition of archaea or selecting against a bacteria-dominated interface (Xia et al., 2017). In brief, the archaeal communities of different cores respond in different ways to specific local controls.

### Environmental History Determines Ecological Context

The sediment cores shared similar biogeochemical parameters, such as sedimentary TOC, and organic matter δ^13^C, δ^15^N and C:N ratios. Repeatedly, studies of uncultured microbes in the sedimentary subsurface tried to correlate community composition with a wide range of biogeochemical or thermal parameters, in the hope that these linkages provide insights into habitat preference and ecophysiology of uncultured archaea (Lazar et al., 2015; McKay et al., 2016; Durbin and Teske, 2012). Although this strategy can yield valuable results, we caution that patterns of archaeal community composition are not deterministically linked to biogeochemical parameters alone, rather, the full context of an ecological interpretation requires that biological and geochemical observations are integrated with the environmental setting and history of a site. For example, the lighter δ^13^C values of sedimentary organic matter in RNVP11 (trending toward −22‰ compared with most values clustering between 20‰ and 21‰), the slightly elevated C:N ratios at this site, increased Si concentrations at depth, or the elevated methane content superimposed on seawater-like porewater characteristics are not in themselves critical factors that determine biological metrics in this core; these factors are significant because they reveal a depositional history of organic-rich sediments overprinted by relatively recent hydrothermalism and methane flux that has left its footprint on the present-day archaeal community. In another example, the archaeal communities of cores SeepP06 and OMZP12 would be assumed to be similar, since both sites are rich in sulfide, methane, and DIC and show rapid sulfate depletion concomitant with methane accumulation; these characteristics indicate strongly reducing marine seep-like conditions with microbial methane and sulfur cycling by functionally equivalent microbial communities. However, the distinct environmental settings and histories of these two cores, at the heavily compacted, seep-influenced base of the Sonora Margin (SeepP06), and under the persistent oxygen minimum zone waters of the upper Sonora Margin (OMZP12), ultimately select for different archaeal communities.

### Conclusion

In the greater Guaymas Basin and Sonora Margin area, complex geological and oceanographic processes impose environmental controls on different sedimentary habitats and their archaeal populations relative to background control sites. In background sediments, archaeal communities vary little with depth after the surface/subsurface transition; here, subsurface communities result primarily from long-term survival likely conferred by relatively reduced mortality (Kirkpatrick et al., 2019). In contrast, localized factors, including water column anoxia, methane seepage, and hydrothermal circulation, constrain the biodiversity and potential biogeochemical activity of sedimentary Archaea across our benthic survey in specific ways. Overall, our observations suggest that local sediment biogeochemistry should be viewed in a broader context—within the history and evolution of a particular site—to reveal its influence on selective survival for certain lineages and subsequent shaping of the resident archaeal community.

### Limitations of the Study

The compositional nature of amplicon-based sequence studies precludes discussion of absolute abundances. Piston coring disturbs small-scale structures near the water-sediment interface; thus, the potential effect of bioturbation on surficial sediment redox state is not addressed in this study.

### Resource Availability

#### Lead Contact

Gustavo A. Ramírez: ramirezg@westernu.edu.

#### Materials Availability

This study did not generate new unique reagents.

#### Data and Code Availability

All sequence data are publicly available at the following repository: NCBI under BioProject PRJNA553578 and accession numbers SRX6444849–SRX6444877.

Geochemical data are available at the BCO-DCO under these reference links:

Porewater methane data: https://www.bco-dmo.org/dataset/661750/data.

Porewater sulfate data: https://www.bco-dmo.org/dataset/661775/data.

Porewater DIC data: https://www.bco-dmo.org/dataset/661658/data.

Porewater sulfide: https://www.bco-dmo.org/dataset/661808/data.

## Methods

All methods can be found in the accompanying Transparent Methods supplemental file.

## Supplemental Information

Document S1. Transparent Methods, Figures S1 and S2, and Tables S1–S4
