# Supplementary material for: The Guaymas Basin Subseafloor Sedimentary Archaeome Reflects Complex Environmental Histories
Source: iScience. 2020 Aug 15;23(9):101459. doi: 10.1016/j.isci.2020.101459 (PMC7476861; doi:10.1016/j.isci.2020.101459)
Supplement: Document S1. Transparent Methods, Figures S1 and S2, and Tables S1–S4 [file mmc1.pdf]

iScience, Volume *23*

Supplemental Information

The Guaymas Basin Subseafloor Sedimentary Archaeome Reflects Complex Environmental Histories

Gustavo A. Ramírez, Luke J. McKay, Matthew W. Fields, Andrew Buckley, Carlos Mortera, Christian Hensen, Ana Christina Ravelo, and Andreas P. Teske

Supplemental Materials

The Guaymas Basin Subseafloor Sedimentary Archaeome Reflects Complex Environmental Histories

Gustavo A. Ramírez[1,2*], Luke J. McKay[3,4], Matthew W. Fields[4,5], Andrew Buckley[1], Carlos Mortera[6], Christian Hensen[7], Ana Christina Ravelo[8] & Andreas P. Teske[1]

Author affiliations:
1: Department of Marine Sciences, University of North Carolina at Chapel Hill, NC, USA.
2: College of Veterinary Medicine, Western University of Health Sciences, Pomona, CA, USA.
3: Department of Land Resources and Environmental Sciences, Montana State University, Bozeman, MT, USA.
4: Center for Biofilm Engineering, Montana State University, Bozeman, MT, USA.
5: Department of Microbiology and Immunology, Montana State University, Bozeman, MT, USA.
6: Instituto de Geofisica, Universidad Nacional Autónoma de México, Coyoacán, Mexico.
7: GEOMAR Helmholtz Centre for Ocean Research, Kiel, Germany.
8: Ocean Sciences Department, University of California, Santa Cruz, CA, USA.

*: Corresponding author

Correspondence:
Dr. Gustavo A. Ramírez
gara1985@email.unc.edu
gramirez@westernu.edu
zombiephylotype@gmail.com

Transparent Methods

Sample Collection. All samples were collected using piston coring during R/V *El Puma* (Universidad Nacional Autónoma de México, UNAM) Expedition Guaymas14 to the Gulf of California, October 14-27th, 2014. A 5-m long piston core (RNVP11) was obtained on Oct 21, 2014 from the central basin within the ring (27°N30.5090/111°W40.6860, 1749 m; core length 4.9 m), parallel to a control core (ContP10) approx. 1 mile to the west of Ringvent (27°N30.5193/111°W42.1722; 1731 m depth, 3.93 m core length) collected on the same day. Core SeepP06 was obtained on Oct. 19 from the lower Sonora Margin, near its boundary with the Ridge flanks (27°N38.8367/111°W36.8595; 1681 m depth, 3.95 m core length). Core OMZP12 was taken on Oct. 22 from the upper Sonora Margin (27°N52.1129/111°W41.5902, 667 m, 4 m core length) in the oxygen minimum zone as previously determined by water column oxygen profiling (Calvert 1964). Core ContP03 was collected on Oct. 17 from the northwestern end of the ridge flanks (27°N37.6759/ 111°W52.5740; 1611 m depth, 3.27 m core length. Core ContP13 was obtained on Oct. 22 from the southeastern ridge flank of Guaymas Basin (27°N12.4470/111°W13.7735, 1859m depth, 3.31 m core length).

Geochemical Analyses. Porewater was obtained from freshly collected sediments on RV *El Puma* by centrifuging ca. 40 ml sediment samples in 50 ml conical Falcon tubes for ca. 5 to 10 minutes, using a Centra CL-2 Tabletop centrifuge (Thermo Scientific) at 1000*g*, until the sediment had settled and produced ca. 8 to 10 ml of porewater. Porewater was extracted from 5 cm thick sediment samples, which are designated by the top of each sample. For example, a "95 cm" geochemistry sample extends from 95 to 100 cm below the sediment surface. Filtered, unamended, porewater samples prepared shipboard were stored at 4°C for shored-based analyses. Sulfate, sulfide, methane, and DIC porewater profiles for cores SeepP06, ContP10, RNVP11, and OMZP12 were previously published (Teske et al 2019), and are re-plotted here for comparison with unpublished profiles from cores ContP03 and ContP13. Porewater analyses were performed as previously described, using the colorimetric Cline assay for sulfide, ion chromatography for sulfate, and GC-IRMS for DIC and methane (Teske et al 2019). Carbon and nitrogen isotopic and elemental composition was determined at the Stable Isotope Laboratory (SIL) at the University of California, Santa Cruz (UCSC). Bulk sediment $\delta^{15}N$ and elemental ratio data were collected using 20 mg samples in Sn capsules; organic $\delta^{13}C$ and elemental composition data were collected using 2.5 mg samples of acidified sediment in

Sn capsules. All samples were measured by Dumas combustion performed on a Carlo Erba 1108 elemental analyzer coupled to a ThermoFinnigan Delt Plus XP isotope ratio mass spectrometer (EA-IRMS). An in-house gelatin standard, Acetanilide, and an in-house bulk sediment standard, "Monterey Bay Sediment Standard", were used in all runs. Reproducibility of an in-house matrix-matched sediment standard is <0.1‰ VPDB for $\delta^{13}C$ and <0.2‰ AIR for $\delta^{15}N$. Data is corrected for blank, and for drift when appropriate. Carbon and nitrogen elemental composition was estimated based on standards of known composition, for which analytical precision is determined to be better than 1 %. Filtered but unamended porewater samples, stored at 4°C, were used for quantifying multiple stable ions, including silicate, by ion chromatography at GEOMAR, Kiel, Germany (Hensen et al 2007). All geochemical data in this study are publicly available at the Biological and Chemical Oceanography Data Management Office (BCO-DMO) under the following dataset IDs: 661750, 661658, 66175 and 661808 for methane, DIC, sulfate and sulfide, respectively.

3. DNA extraction and gene sequencing

Samples for DNA sequencing [approx. 2 cm^3 each] were obtained by syringe coring at the indicated depth [in cm] below the sediment surface. Freshly collected samples were immediately frozen (-80°C) for storage and transport back to shore. DNA for all survey sites was extracted from ~0.5-1.0 cm^3 sediment sample volumes using the Powersoil DNA extraction kit according to the manufacturer's instructions (QIAGEN, Carlsbad, CA, USA). Archaeal 16S rRNA gene amplicons from DNA extracts were generated using the following primer set: A751F: 5'-CGA CGG TGA GRG RYG AA-3' and A1204R: 5'-TTM GGG GCA TRC NKA CCT-3'using the following thermocycling program: initial denaturation for 2 mins at 94°C, 30 x [94°C for 1 min, 55°C for 1 min, 72°C for 1 min], and a final 10 min extension at 72°C, as suggested elsewhere (Baker et al 2003). Amplicons were sequenced on an Illumina MiSeq platform (Illumina, San Diego, CA, USA) at the Center for Biofilm Engineering in Bozeman, Montana. Sequencing run specifications are found in the Visualization and Analysis of Microbial Population Structures (VAMPSs) website (https://vamps.mbl.edu/resources/primers.php) (Huse et al 2014).

4. Sequence Processing

Sequences were processed with *mothur* v.1.39.5 (Schloss et al 2009) following the *mothur* Illumina MiSeq SOP (Kozich et al 2013). Briefly, forward and reverse reads were merged into

contigs and selected based on primer-specific amplicon length and the following parameters: maximum homopolymers of 6bp, and zero ambiguities. High quality sequences were aligned against the *mothur*-recreated Silva SEED v132 database (Yarza et al 2010) and subsequently pre-clustered at 1% dissimilarity. As suggested elsewhere (Kozich et al 2013), spurious sequences are mitigated by abundance ranking and merging with rare sequences based on minimum differences of three base pairs. Chimeras were detected and removed using UCHIME de novo mode (Edgar et al 2011). Sequences were then clustered, by generating a distance matrix using the average neighbor method, into operational taxonomic units (OTUs, 97% similarity cutoff). OTU classification was performed on *mothur* using the SILVA v132 database as implemented using the classify.seqs command using the Wang algorithm (kmer assignment with 1/8 kmer replacement as bootstrap) and cutoff=80 (minimal bootstrap value for sequence taxonomy assignment). All sequence data are publically available at the following repository: NCBI under BioProject PRJNA553578 and accession numbers SRX6444849-SRX6444877.

5. Sequence Analyses

5.1 Community Analyses and Visualizations

Community analyses were performed in *RStudio* version 0.98.1091 (Racine 2012), implemented in R version 3.5.2, using the *vegan* (Oksanen et al 2015) and *phyloseq* (McMurdie and Holmes 2013) R-packages. Sample richness analyses used the R package *breakaway* (Willis et al. 2017) for inferring precision of diversity estimations given the heterologous sequencing depth. Data were rlog normalized using *DESeq2* (Love et al 2014) prior to ordination using Bray-Curtis distances. An identical normalization strategy was used on Bray-Curtis distances for co-occurrence network analysis performed using the makenetwork() *phyloseq* command and visualized using the *igraph* R-package. DESeq2 was also used to perform differential abundance analyses of taxa with low abundance taxa ($n < 100$ total reads per OTU) removed for the un-rarefied dataset, as suggested elsewhere (McMurdie and Holmes 2014).

5.2 Phylogenetic Analyses

Sequence alignments were performed using the high speed multiple sequence alignment program MAFFT (Katoh and Standley 2013) with the command: mafft --maxiterate 1000 –localpair seqs.fasta > aligned.seqs.fasta. Maximum likelihood trees with 100 bootstrap support were constructed using the RAxML (Stamatakis 2014) program using the following

parameters: raxmlHPC -f a -m GTRGAMMA -p 12345 -x 12345 -# 100 -s aligned.seqs.fasta -n T.tree, -T 4 ML search + bootstrapping. Newick trees files were uploaded to FigTree v1.4.2 for visualization.

Core ID	Latitude	Longitude	Collection Date (2014)	Core Length (m)	Water Depth (m)
ContP3	27°N37.6759	111°W52.5740	Oct. 17	3.27	1611
SeepP6	27°N38.8367	111°W36.8595	Oct. 19	3.95	1681
ContP10	27°N30.5193	111°W42.1722	Oct. 21	3.93	1731
RNVP11	27°N30.5090	111°W40.6860	Oct. 21	4.9	1749
OMZP12	27°N52.1129	111°W41.5902	Oct. 22	4	667
ContP13	27°N12.4470	111°W13.7735	Oct. 22	3.31	1859

Table S1. Related to Figure 1. Core site metadata.

Sample	Lokiarchaea
ContP03_9	0.019%
ContP03_104	0.007%
ContP03_202	0.000%
ContP03_301	0.000%
SeepP06_5	0.024%
SeepP06_105	0.009%
SeepP06_205	0.000%
SeepP06_304	0.000%
SeepP06_394	0.000%
ContP10_5	0.012%
ContP10_104	0.000%
ContP10_204	0.000%
ContP10_303	0.000%
ContP10_378	0.000%
RNVP11_5	0.000%
RNVP11_95	0.020%
RNVP11_195	0.000%
RNVP11_295	0.000%
RNVP11_394	0.000%
RNVP11_486	0.000%
OMZP12_5	0.003%
OMZP12_105	0.000%
OMZP12_204	0.000%
OMZP12_304	0.000%
OMZP12_379	0.003%
ContP13_5	0.006%
ContP13_111	0.002%
ContP13_210	0.000%
ContP13_310	0.000%

Table S2. Related to Figure 6. Percent of total community contribution of Lokiarchaea sequences in all samples based on SILVA132 taxonomic assignments.

Core_cmbsf	All_ANME	ANME-1	ANME-2a-2b	ANME-2c
ContP3_009	0.034	0.000	0.000	0.034
ContP3_104	0.002	0.002	0.000	0.000
ContP3_202	0.000	0.000	0.000	0.000
ContP3_301	0.000	0.000	0.000	0.000
SeepP6_005	0.030	0.018	0.012	0.000
SeepP6_105	8.863	8.863	0.000	0.000
SeepP6_205	32.063	32.063	0.000	0.000
SeepP6_304	32.446	32.440	0.006	0.000
SeepP6_394	39.810	39.810	0.000	0.000
ContP10_005	0.111	0.088	0.024	0.000
ContP10_104	0.092	0.092	0.000	0.000
ContP10_204	0.003	0.000	0.003	0.000
ContP10_303	0.447	0.447	0.000	0.000
ContP10_378	0.000	0.000	0.000	0.000
RNVP11_005	0.009	0.009	0.000	0.000
RNVP11_095	0.000	0.000	0.000	0.000
RNVP11_195	0.988	0.988	0.000	0.000
RNVP11_295	0.000	0.000	0.000	0.000
RNVP11_394	0.000	0.000	0.000	0.000
RNVP11_486	0.000	0.000	0.000	0.000
OMZP12_005	0.000	0.000	0.000	0.000
OMZP12_105	0.123	0.121	0.002	0.000
OMZP12_204	2.098	2.098	0.000	0.000
OMZP12_304	0.629	0.629	0.000	0.000
OMZP12_379	0.967	0.967	0.000	0.000
ContP13_005	0.476	0.429	0.029	0.018
ContP13_111	0.006	0.002	0.004	0.000
ContP13_211	0.055	0.012	0.043	0.000
ContP13_310	0.004	0.000	0.004	0.000

Table S3. Related to Figure 6. Percent of total community contribution of ANME sequences in all samples based on SILVA132 taxonomic assignments. The All_ANME column shows the percent contribution of sequences classified as ANME in each sample. Columns ANME-1, ANME-2a-2b, and ANME-2c show the percent breakdown of the respective ANME lineages in each sample and their sum is equal to the All_ANME column percentage.

Sample Name	DNA yield (ng/µL)	Num. of seqs post Mothur QC and chimera removal
ContP3_9	7	21,443
ContP3_104	6.9	47,239
ContP3_202	6.6	16,038
ContP3_301	9.4	45,559
SeepP6_5	9	17,196
SeepP6_105	4.3	11,595
SeepP6_205	9.1	9,274
SeepP6_304	9.4	18,043
SeepP6_394	8	10,047
ContP10_5	9.2	25,975
ContP10_104	7.7	12,289
ContP10_204	8	35,076
ContP10_303	14.5	29,782
ContP10_378	7.6	25,682
RNVP11_5	6.7	11,184
RNVP11_95	6.7	30,452
RNVP11_195	7.1	2,978
RNVP11_295	7	19,515
RNVP11_394	7.4	14,142
RNVP11_468	7.9	29,851
OMZP12_5	7.9	63,690
OMZP12_105	9	51,384
OMZP12_204	7.8	167,234
OMZP12_304	7.3	154,763
OMZP12_379	8.1	76,729
ContP13_5	6.6	17,573
ContP13_111	7.9	47,432
ContP13_210	6.8	25,989
ContP13_310	7.3	24,873

Table S4. Related to Figure 3. Total DNA yield and high-quality sequence numbers for all samples.

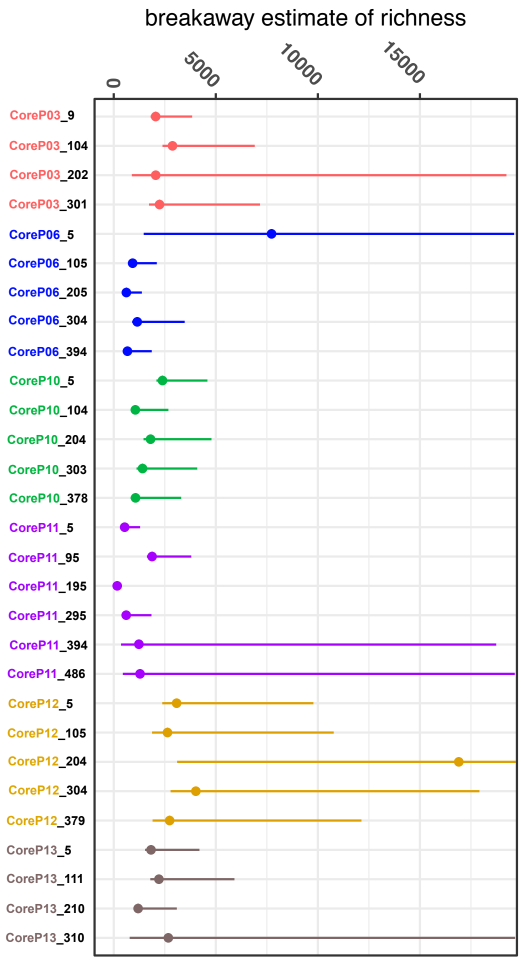

Figure S1. Related to Figure 3. Breakaway estimate of total species richness with model confidence intervals for color-coded cored site for all depths.

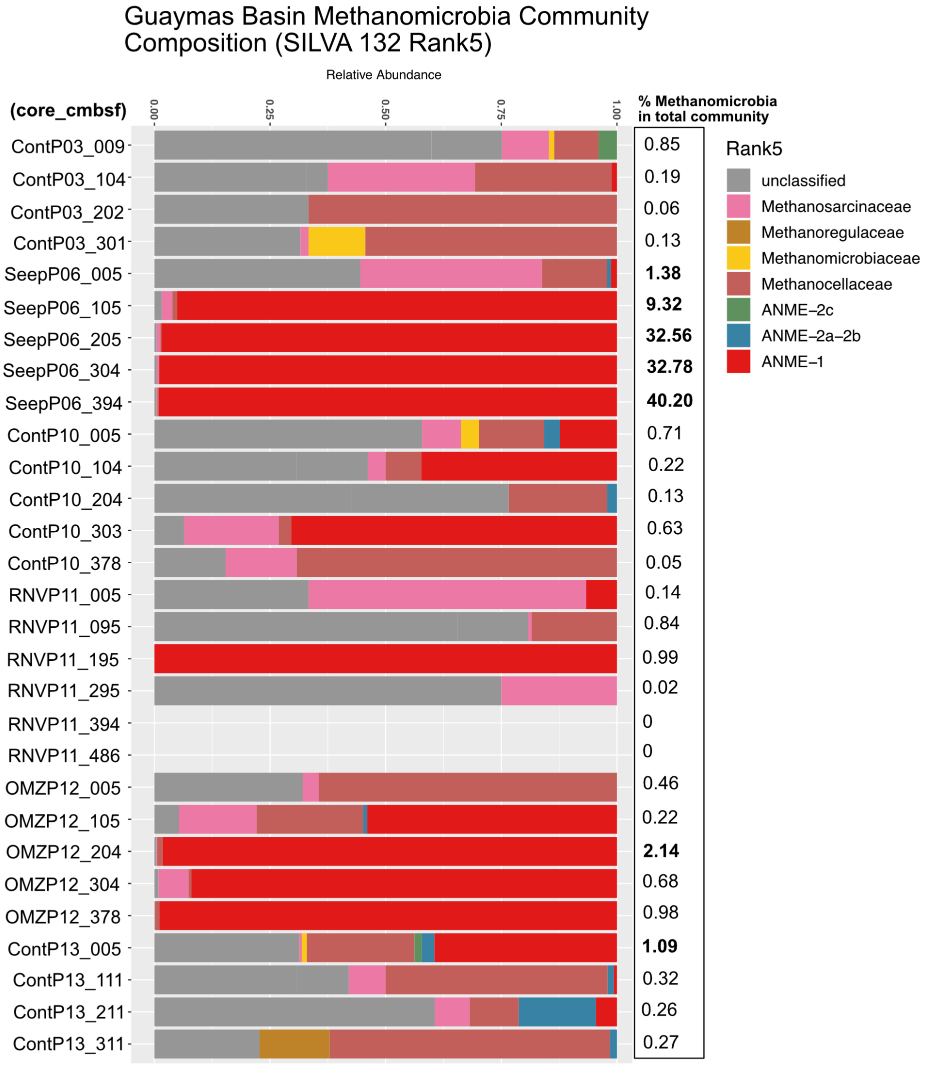

Figure S2. Related to Figure 6. Methanomicrobia community composition for all cores in this survey.

Works Cited

Baker GC, Smith JJ, Cowan DA (2003). Review and re-analysis of domain-specific 16S primers. *J of Microbiol Meth* **55:** 541-555.

Calvert SE (1964). "Factors affecting distribution of laminated diatomaceous sediments in the Gulf of California" In Marine Geology of the Gulf of California, Vol. 3, eds. T.H. van Andel and G.G. *Shor, (Tulsa: American Association of Petroleum Geologists Memorir)* **3:** 311-330.

Edgar RC, Haas BJ, Clemente JC, Quince C, Knight R (2011). UCHIME improves sensitivity and speed of chimera detection. *Bioinformatics* **27:** 2194-2200.

Hensen C, Nuzzo M, Hornibrook E, Pinheiro LM, Bock B, Magalhães VH *et al* (2007). Sources of mud volcano fluids in the Gulf of Cadiz—indications for hydrothermal imprint. *Geochim et Cosmochim Acta* **71:** 1232-1248.

Huse SM, Mark Welch D, Voorhis A, Shipunova A, Morrison HG, Eren AM *et al* (2014). VAMPS: a website for visualization and analysis of microbial population structures. *BMC Bioinformatics* **15**.

Katoh K, Standley DM (2013). MAFFT multiple sequence alignment software version 7: improvements in performance and usability. *Mol Biol Evol* **30:** 772-780.

Kozich JJ, Westcott SL, Baxter NT, Highlander SK, Schloss PD (2013). Development of a dual-index sequencing strategy and curation pipeline for analyzing amplicon sequence data on the MiSeq Illumina sequencing platform. *Appl Environ Microbiol* **79:** 5112-5120.

Love MI, Huber W, Anders S (2014). Moderated estimation of fold change and dispersion for RNA-seq data with DESeq2. *Genome Biol* **15:** 550.

McMurdie PJ, Holmes S (2013). phyloseq: an R package for reproducible interactive analysis and graphics of microbiome census data. *PLoS One* **8:** e61217.

McMurdie PJ, Holmes S (2014). Waste not, want not: why rarefying microbiome data is inadmissible. *PLoS Comput Biol* **10**.

Oksanen J, Blanchet FG, Kindt R, Legendre P, Minchin PR, O'Hara RB *et al* (2015). vegan: Community Ecology Package. R Package Version 2.2-1. *Available online at: http://CRANR-projectorg/package=vegan*.

Racine JS (2012). RStudio: A Platform-Independent IDE for R and Sweave. *Journal of Applied Econometrics* **27:** 167-172.

Schloss PD, Westcott SL, Ryabin T, Hall JR, Hartmann M, Hollister EB *et al* (2009). Introducing mothur: open-source, platform-independent, community-supported software for describing and comparing microbial communities. *Appl Environ Microbiol* **75:** 7537-7541.

Stamatakis A (2014). RAxML version 8: a tool for phylogenetic analysis and post-analysis of large phylogenies. *Bioinformatics* **30:** 1312-1313.

Teske A, McKay L, Ravelo AC, Aiello I, Mortera C, Núñez-Useche F *et al* (2019). Characteristics and evolution of sill-driven off-axis hydrothermalism in Guaymas Basin- the Ringvent site. *Scientific Reports* **9**.

Yarza P, Ludwig W, Euzeby J, Amann R, Schleifer KH, Glöckner FO *et al* (2010). Update of the All-Species Living Tree Project based on 16S and 23S rRNA sequence analyses. *Syst Appl Microbiol* **33:** 291-299.
